# Transcriptomic Insight into Underground Floral Differentiation in *Erythronium japonicum*

**DOI:** 10.1155/2022/4447472

**Published:** 2022-01-18

**Authors:** Hongtao Wang, Lifan Zhang, Peng Shen, Xuelian Liu, Rengui Zhao, Junyi Zhu

**Affiliations:** ^1^College of Agriculture, Jilin Agricultural University, China; ^2^School of Life Sciences, Tonghua Teachers College, China

## Abstract

*Erythronium japonicum* Decne (Liliaceae) flowers in early spring after overwintering. Its sexual reproduction process includes an underground development process of floral organs, but the underlying molecular mechanisms are obscure. The present study is aimed at exploring the transcriptional changes and key genes involved at underground floral developmental stages, including flower primordium differentiation, perianth differentiation, stamen differentiation, and pistil differentiation in *E. japonicum*. Multistage high-quality transcriptomic data resulted in identifying putative candidate genes for underground floral differentiation in *E. japonicum.* A total of 174,408 unigenes were identified, 28,508 of which were differentially expressed genes (DEGs) at different floral developmental stages, while only 44 genes were identified with conserved regulation between different stages. Further annotation of DEGs resulted in the identification of 270 DEGs specific to floral differentiation. In addition, *ELF3*, *PHD*, *cullin 1*, *SE14*, *ZSWIM3*, *GIGNATEA*, and *SERPIN B* were identified as potential candidate genes involved in the regulation of floral differentiation. Besides, we explored transcription factors with differential regulation at different developmental stages and identified *bHLH*, *FAR1*, *mTERF*, *MYB-related*, *NAC*, *Tify*, and *WRKY* TFs for their potential involvement in the underground floral differentiation process. Together, these results laid the foundation for future molecular works to improve our understanding of the underground floral differentiation process and its genetic regulation in *E. japonicum.*

## 1. Background

*Erythronium japonicum* Decne (Liliaceae) is a spring ephemeral plant commonly known as the Asian fawn lily [1]. The known geographic origins of *E. japonicum* are Northeast China, Japan, and Korea [1, 2]. *E. japonicum* produces an eye-appealing florescence with reddish-purple flowers [3]. Its vernal characteristics and florescence in early spring make it a perfect ornamental plant. In ephemeral spring plants, flower buds are usually initiated before dormancy induction and continue during the dormancy period [4]. Many studies have been conducted to understand the life cycle, growth habits, reproduction, morphological distinctions, and environmental dynamics in *E. japonicum* [2, 3, 5–8]. However, there is an apparent lack of studies concerning the molecular mechanisms underlying the underground floral differentiation in *E. japonicum*.

The floral structures are originated in the floral primordium; however, the specific differentiation of stamens and pistils governs the further floral development [9]. Therefore, it is important to understand the regulatory pathways underlying floral differentiation. Floral differentiation has been widely studied in many plant species viz., *Jatropha curcas* [9], *Brassica napus* [10], *Camellia sinensis* [11], *Populus* [12] *Dianthus caryophyllus* [13], *Litsea cubeba* [14], *Rosa chinensis* [15], *Lilium* [16], and *Juglans regia* [17]. In Arabidopsis, multiple pathways have been identified responsible for floral differentiation including, gibberellic acid (GA), vernalization pathways, aging pathway, and sugar signaling pathway [18–20]. FLOWERING LOCUS T (*FT*) is the integral component in flowering regulation, and most of the flowering-related pathways converge to *FT* regulation [11]. Regulation of flowering is a complex mechanism and is generally triggered by environmental variables, i.e., temperature and humidity [21]. Furthermore, an overlap between pathways governing flowering and dormancy has been reported [21]. For instance, FLOWERING LOCUS C (*FLC*) and FRIGIDA (*FRI*) have been reported with reduced expression during vernalization [22, 23]. The considerable overlap between flowering and dormancy needs to be explored further to exploit their regulation.

Advances in omics have increased our understanding of complex mechanisms regulating plant growth and development [24–29]. Multiple approaches including, transcriptomics [30–32], genomics [33, 34], phenomics [35, 36], and proteomics [37, 38] have been used for uncovering flowering mechanism in plants. RETARDED PALEA1 [39], MADS-box [40], SDRLK, PEBP [41], FLOWERING LOCUS C (FLC) [42], SHORT VEGETATIVE PHASE (SVP), FLOWERING LOCUS M (FLM) [42], LEAFY (LFY) [43], and APETALA1 (AP1) [44] have been previously identified for their subtle role in the regulation of flowering in different plants species. The characterization of these genes through targeted approaches is complemented by high-throughput technologies [30].

This study investigated the transcriptional changes during floral differentiation in *E. japonicum* at four developmental stages viz., flower primordium differentiation, perianth differentiation, stamen differentiation, and the pistil differentiation period. Our analysis of underground the floral differentiation in *E. japonicum* provides an overview of differentially expressed genes and their roles in developing flower organs after overwintering.

## 2. Methods

### 2.1. Plant Materials and Sample Collection

**Figure 1:**
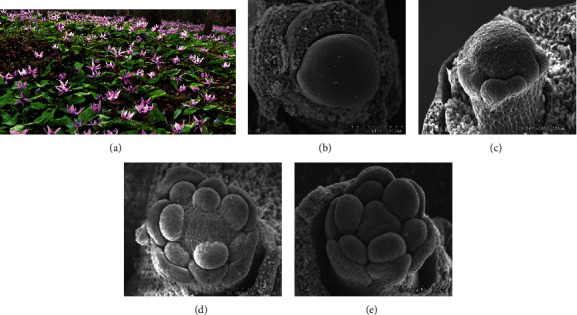
A pictorial description of *E. japonicum*. (a) Flowering phase. (b) Microscopic view of flower primordium differentiation stage. (c) Microscopic view of perianth differentiation stage. (d) Microscopic view of stamen differentiation stage. (e) Microscopic view of pistil differentiation stage.

The study area includes Tuodaoling region (125°55′45^″^ ~125°35′59^″^E, 41°37′55^″^ ~41°37′59^″^N) between Laoling Mountains and Longgang Mountains in Tonghua Section of Changbai Mountains in Northeast China (700~750 m above sea level). Sampling time was determined according to plant growth and development. After the above-ground parts of the population withered and died in late May, meristem samples in bulbs were collected at four stages of underground flower organ development, including flower primordium differentiation (from May 28th to June 9th), perianth differentiation (from June 9th to June 17th), and stamen differentiation (from June 15th to June 20th) and the pistil differentiation period (from June 17th to June 30th), according to a previously published report [45]. The sample collection stages have been elaborated in Figure 1. The samples of *E. japonicum* were collected in three biological replicates from different plants. During the differentiation period, samples were collected from the under-forest plot every ten days. After collecting the floral organ meristem samples, samples were wrapped in aluminum foil and frozen in liquid nitrogen immediately. Later, the samples were stored in a refrigerator at -80°C until further use. During sampling, the phenological phase of each plant was recorded. A total of 12 samples were used for transcriptome sequencing analysis. All samples were obtained from the wild, and no permissions are necessary to collect such samples. The formal identification of the samples was conducted by Prof Rengui Zhao, and novoucher specimens have been deposited.

### 2.2. RNA Extraction, Library Preparation, and Sequencing

Transcriptome sequencing was performed by constructing four libraries corresponding randomly collected flower bud samples, each with three replicates. Total RNA was extracted using TRIzol reagent (TaKaRa, China). To access, the quality of extracted RNA contamination and RNA integrity number was checked using 1% agarose gel and Agilent 2100 Bioanalyzer system (Agilent Technologies, CA, USA), respectively. Pair end sequencing libraries were constructed using 3 *μ*g RNA for each sample. Further, libraries were generated using NEBNext® UltraTM RNA Library Prep Kit for Illumina® (NEB, USA) following the manufacturer's instructions. Illumina HiSeq platform was utilized for RNS sequencing and was performed by company Novogene (https://en.novogene.com/). Following, the libraries were sequenced by paired-end sequencing on Illumina Hiseq.

Low-quality reads and short sequence reads (<50 bp) were removed using FastQC and in-house Perl scripts. Finally, clean reads were de novo assembled using Trinity v.2.6.6 [46].

### 2.3. Gene Expression Quantification and Differential Expression Analysis

The mapped reads numbers were calculated using featureCounts v1.5.0-p3 [47]. Then, calculating the expected number of fragments per kilobase of exon model per million reads mapped (FPKM) of each gene based on the length of each gene and reads count mapped to the gene. Differentially expressed genes (DEGs) between samples from different floral developmental stages were identified using the DESeq R package (v1.18.0) [48]. The false discovery rate (FDR) method was used to estimate the *p* value threshold in multiple tests to judge the significance of gene expression. When FDR ≤ 0.05 and FPKM values showed at least 2-fold difference among samples, the gene was considered DEG. Conserved DEGs across developmental stages were identified InteractiVenn [49]. The DEGs were classified using GO [50] and KEGG enrichment analysis [51]. The annotated DEGs were further screened for their functions related to floral bud differentiation, and their corresponding expression levels at different stages were compared.

Furthermore, transcription factors were identified using iTAK by integrating PlnTFDB and PlantTFDB [52]. The principle is to identify TF by hmmscan comparison by using the TF family information.

### 2.4. Gene Expression Validation Using qRT-PCR

Quantitative real-time PCR (qRT-PCR) was performed for selected genes to verify the transcriptomic data and their corresponding gene expressions and different floral differentiation stages. Tiangen RNAprep Pure Plant kit (Tiangen biotech., Beijing, China) was used to isolate total RNA from samples. Eighteen genes related to floral differentiation and flowering-related pathways were selected, and corresponding primers were designed for qRT-PCR using the Oligo-7 software (Table S1). The primers were synthesized by Sangon Biotech (Shanghai, China). The cDNA was extracted from RNA and used as a template to make the reaction for qRT-PCR by using Takara qPCR kit SYBR Premix Ex TaqTM II (Tli RNaseH Plus). Three biological repeats were used for each qRT-PCR reaction and analysis was performed using 2^−*ΔΔ*Ct^ method [53].

## 3. Results

**Figure 2:**
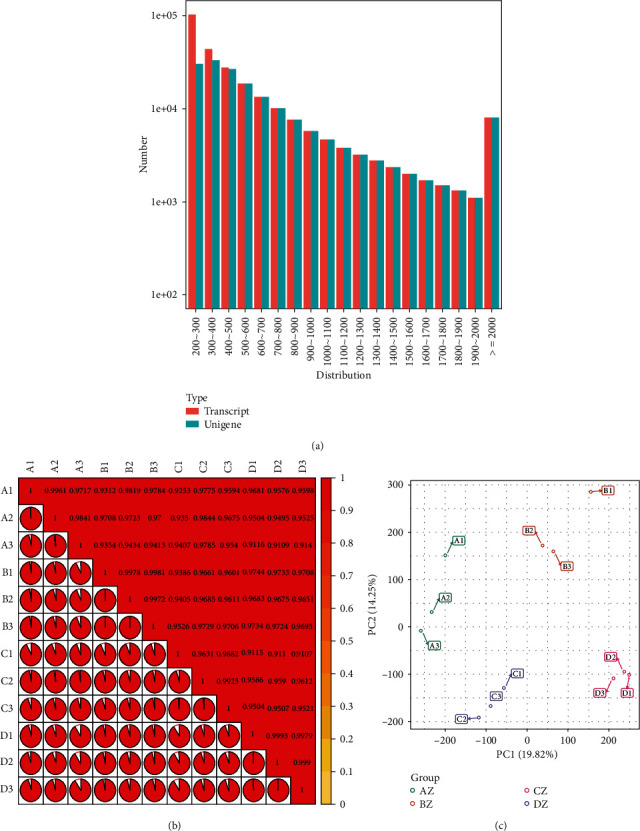
(a) Sequence length distribution for transcripts and unigenes. (b) Correlation analysis of FPKM values, (c) PCA graph representing the distribution of different samples based on their corresponding FPKM values ∗A, B, C, and D in the figure correspond to four floral differentiation stages: viz., flower primordium differentiation, perianth differentiation, stamen differentiation, and pistil differentiation.

Based on morphological distinction, we divided the floral bud differentiation of *E. japonicum* into four stages, viz., flower primordium differentiation, perianth differentiation, stamen differentiation, and pistil differentiation. Tissue samples from each stage, in three replicates, were subjected to RNA-sequencing. A separate transcriptome from each stage was subsequently analyzed to identify the molecular regulation of floral differentiation in *E. japonicum*. Approximately 548 million raw reads were filtered for clean reads (520 million). After filtering for unqualified reads, 78.04 Gb of clean bases were obtained where Q20% was above 97.38%, and Q30% was above 92.72%. The GC contents ranged from 48.35% to 50.11% (Table S2). After de novo assembly, 263,291 transcripts and 174,408 unigenes were identified with a mean length of 561 and 706, respectively. The spliced transcripts were sorted lengthwise, and N50 distribution was estimated to be 727 (Transcripts) and 880 (Unigenes). The estimated sequence length distribution of all unigene and transcripts has been presented in Figure 2(a). The identified unigenes were annotated against different databases, viz., GO (26.01%), KEGG (24.36%), Swiss-prot (20.62), NR (34.02%), KOG (21.18%), and Pfam (22.0%) [53–55] (Figure S1).

Before proceeding to the comparative analysis of transcriptomes, the individual transcriptome data were analyzed for quantitation. Correlation and principal component analysis (PCA) were performed. All samples showed highly significant correlations (Figure 2(b)), while PCA based on FPKM values depicted uniform distribution of replicates for each sample. However, all four samples were distributed as separate groups, suggesting different transcriptional regulations at each stage (Figure 2(c)).

### 3.1. Differentially Expressed Genes Associated with Floral Bud Differentiation

**Figure 3:**
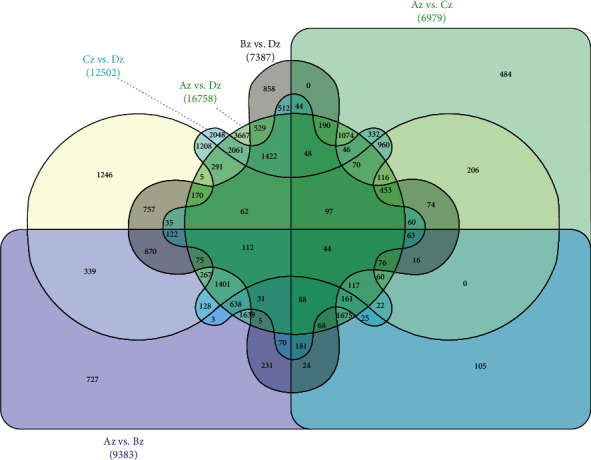
Venn diagram representing common differentially expressed genes between Az vs. Bz, Az vs. Cz, Az vs. Dz, Bz vs. Cz, Bz vs. Dz, and Cz vs. Dz. Where A, B, C, and D in the figure correspond to four floral differentiation stages viz., flower primordium differentiation, perianth differentiation, stamen differentiation, and the pistil differentiation, respectively.

**Figure 4:**
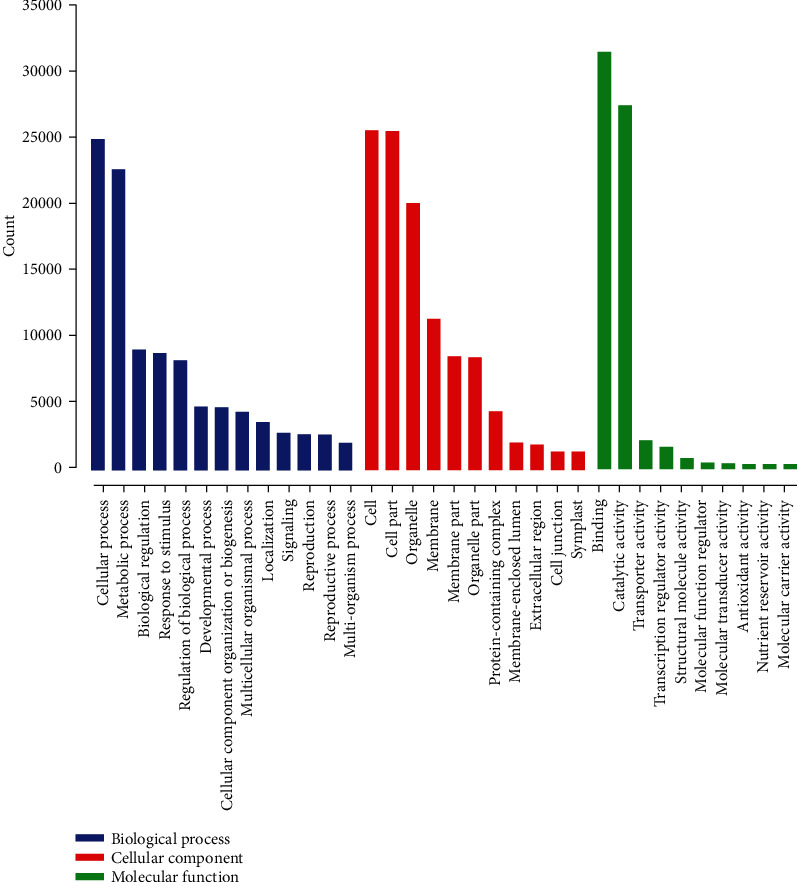
Major GO terms, viz., biological processes, molecular functions, and cellular components associated with the DEGs. The *x*-axis represents the major GO terms, and *y*-axis represents the count number for each GO term.

**Figure 5:**
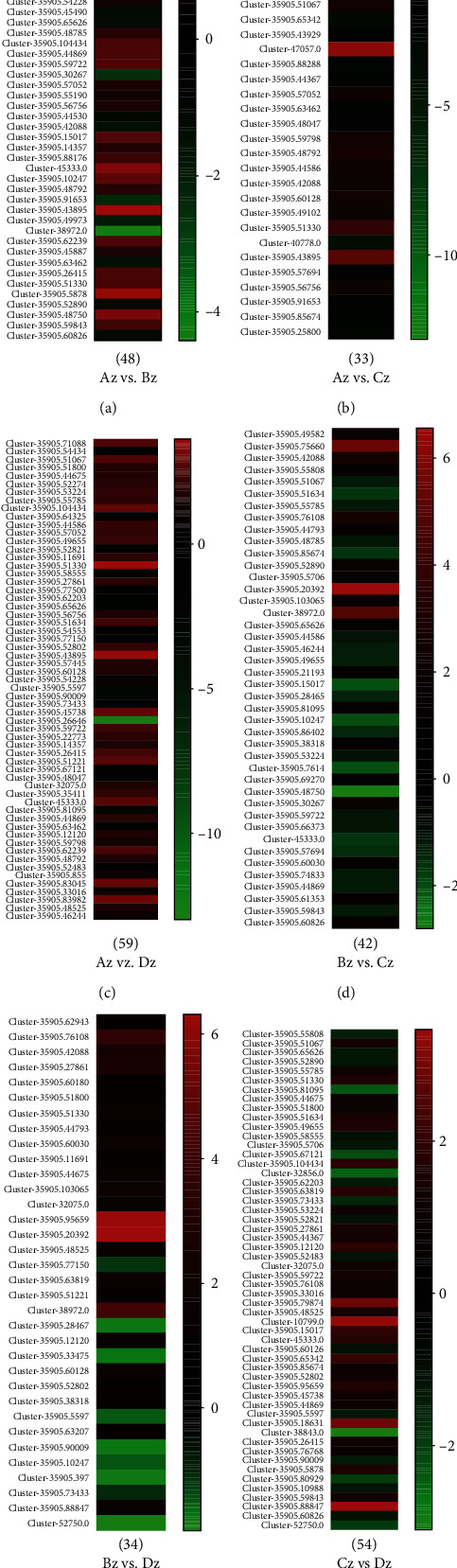
DEGs related to floral differentiation in *E. japonicum* identified through pairwise comparison of four developmental stages. (a) Log_2_FC of 48 DEGs between Az and Bz. (b) Log_2_FC of 33 DEGs between Az and Cz. (c) Log_2_FC of 59 DEGs between Az and Dz. (d) Log_2_FC of 42 DEGs between Bz and Cz. (e) Log_2_FC of 34 DEGs between Bz and Dz. (f) Log_2_FC of 42 DEGs between Cz and Dz. ∗A, B, C, and D in figure correspond to the four floral differentiation stages: viz., flower primordium differentiation, perianth differentiation, stamen differentiation, and pistil differentiation.

A total of 28508 genes were identified as differentially expressed among all floral developmental stages (Table S3). Further, to identify the differentially expressed genes between different stages of floral differentiation, all pairwise comparisons, viz., Az vs. Bz, Az vs. Cz, Az vs. Dz, Bz vs. Cz, Bz vs. Dz, and Cz vs. Dz were explored, and we identified 9,383, 6,979, 16,758, 9,522, 7,387, and 12,502 DEGs, respectively. A total of 44 DEGs (11 upregulated and 33 downregulated) were identified as conserved DEGs across all four floral development stages (Figure 3). Major GO terms associated with DEGs have been presented as Figure 4. Based on GO classifications, 48, 33, 59, 42, 34, and 54 DEGs were identified related to floral differentiation in Az vs. Bz, Az vs. Cz, Az vs. Dz, Bz vs. Cz, Bz vs. Dz, and Cz vs. Dz, respectively (Figure 5, Table S4-S9). Log_2_FC values for the identified DEGs related to floral differentiation have been presented as a heat map in Figure 5. *Cluster-35905.71088*; *EARLY FLOWERING 3* (*ELF 3*), *Cluster-35905.51067*; *PHD finger protein*, *Cluster-35905.55785*; *cullin 1* (*CUL1*), *Cluster-35905.53224*; *lysine-specific demethylase* (*SE14*), *Cluster-35905.49655*; *L-aspartate oxidase*, *Cluster-35905.46244*; *zinc finger SWIM domain-containing protein 3* (*ZSWIM3*), *Cluster-35905.53765*; *callose synthase*, *Cluster-35905.55879*; *RAB6A-GEF complex partner protein 1*, *ribose 5-phosphate isomerase A*, *GIGANTEA*, *Cluster-35905.62239*; *SERPIN B*, and *Cluster-35905.59843*; *flowering locus K homology domain-like isoform X1* were upregulated at perianth differentiation stage, suggesting an active role of these DEGs in the development of flower buds (Table S4). In contrast, *Cluster-35905.60030*; *methyl-CpG-binding domain-containing protein 9* (*AtMBD9*), *Cluster-35905.60826*; *flowering time control protein FY isoform X1*; and *Cluster-38972.0*; *CONSTANS* showed downregulation at the perianth differentiation stage.

*ELF 3* and *FT* (*FLOWERING LOCUST T*), *cullin 1*, *GLP1*, and *CONSTANS* showed significantly higher upregulation at the stamen differentiation stage compared to primordium differentiation and perianth differentiation (Table S5 and Table S7). Interestingly, the number of floral differentiation genes upregulated at pistil differentiation increased significantly. *ELF3*, *CONSTANS*, and cullin1 were upregulated at all floral developmental stages (Table S3-S7). Besides, *BRPF1* (*bromodomain and PHD finger-containing 1*), *HERC4* (*Probable E3 ubiquitin-protein ligase*), *Hsp 70*, *SPA1* (*Protein SUPPRESSOR OF PHYA-105 1*), *ZSWIM3*, and *MYB*-*related transcription factor LHY* showed specific upregulation at the pistil differentiation stage (Table S6, S8, and S9).

### 3.2. Transcription Factors Associated with Floral Differentiation

Understanding the developmental regulatory networks is essential to comprehend the specific developmental process. Transcription factors (TF) play a pivotal role in the developmental process. Therefore, we explored the DEGs and identified 213, 181, 397, 303, 191, and 316 TFs in Az vs. Bz, Az vs. Cz, Az vs. Dz, Bz vs. Cz, Bz vs. Dz, and Cz vs. Dz, respectively (Table S10). AP2/ERF-ERF, bHLH, FAR1, mTERF, MYB-related, NAC, Tify, and WRKY were the most prominent TFs differentially expressed at the different floral differentiation stages. Further annotation of these TFs families identified TFs associated with floral differentiation. Based on corresponding annotation results, 41 TFs were identified (Table S11). The differential expression of these TFs has been presented in Table S10. Twelve TFs, viz., FAR1, EIL, IWS1, B3, DDT, SNF2, PHD, NAC, SWI/SNF-WI3, RWP-RK, PHD, and mTERF exhibited concomitant upregulation at all floral transition stages. While remaining 23 TFs were downregulated at perianth differentiation, stamen differentiation, and the pistil differentiation stages compared to primordium differentiation. The variable transcriptomic landscape of TFs suggested their potential role in floral differentiation in E. japonicum. However, further study is required to confirm the regulatory role of these TFs in the floral development stages.

**Figure 6:**
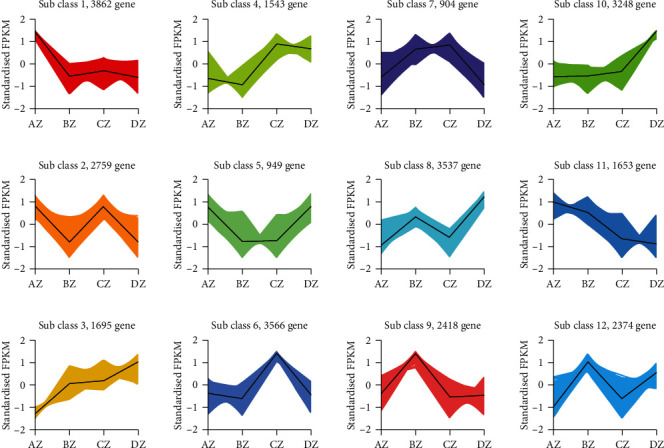
*K*-means clustering of differentially expressed genes based on standardized FPKM values. The numbers of genes clustered in each subclass are mentioned above. ∗Az, Bz, Cz, and Dz in the figure correspond to four floral differentiation stages viz., flower primordium differentiation, perianth differentiation, stamen differentiation, and the pistil differentiation, respectively.

FPKM values of identified 28,508 were subjected to K-mean clustering analysis to identify coexpressed TF genes with DEGs at the four floral development stages (Figure 6). We identified subclasses 1, 3, 6, and 8 containing the most number of structural DEGs related to flower development. These subclasses contains several TFs, including AP2/ERF-ERF, bHLH, FAR1, mTERF, MYB-related, NAC, Tify, and WRKY. Further molecular characterization of identified coexpressed TFs with structural genes can potentially narrow down the DEGs involved in floral differentiation in E. japonicum.

### 3.3. qRT-PCR-Based Verification of Expression Pattern of Identified Genes

**Figure 7:**
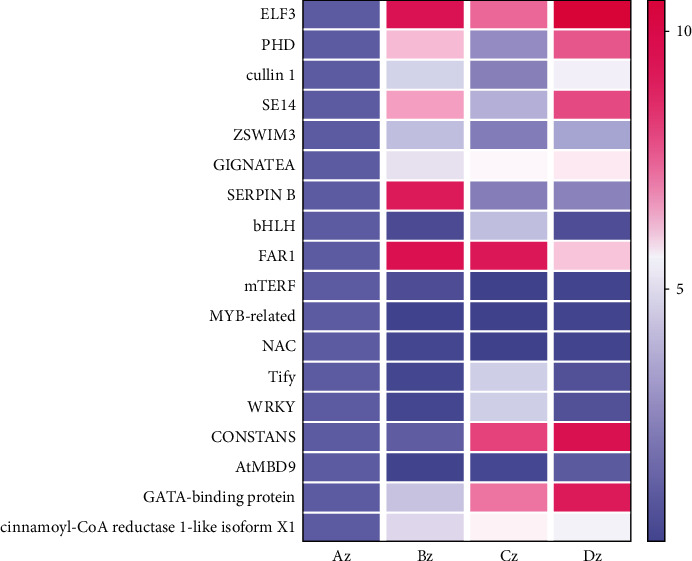
qRT-PCR gene expression profile of selected genes related to floral differentiation at different floral differentiation stages viz., Az (primordium differentiation), Bz (perianth differentiation), Cz (stamen differentiation), and Dz (pistil differentiation). The average relative expression was plotted.

Based on the transcriptome analysis and further bioinformatics analyses, we identify 18 genes potentially associated with floral differentiation in E. japonicum. To validate the transcriptome data and corresponding expression of selected genes at different floral development stages, we performed qRT-PCR-based validation. As a result, the expression profile of selected genes confirmed the transcriptome's reliability and demonstrated the differential expression pattern at the four floral developmental stages (Figure 7).

## 4. Discussion

Floral organ development in ornamental plants is a key process shaping their commercial value [47, 56]. To meet the ever-increasing demand in floriculture industry, many wild flowers have been domesticated for their commercial use [57]. *Erythronium japonicum* Decne (Liliaceae) is one such example that is native to Asia [2]. *E. japonicum* is an early spring ephemeral, and the initial flower development phase starts underground without photoperiod induction and vernalization [58]. Therefore, it is important to understand and explore the floral differentiation process in *E. japonicum* to better utilize its commercial value. The present study is aimed at exploring diverse transcriptomic landscape pertaining to different developmental stages involved in floral morphogenesis, viz., including primordium differentiation, perianth differentiation, stamen differentiation, and pistil differentiation stage.

Floral induction is a series of developmental processes, with each stage providing substantial inputs to govern the overall process [59]. Based on a previous report [45], we selected four stages of underground flower development in *E. japonicum*, and samples from each stage were subjected to transcriptomic profiling. A similar approach for exploring transcriptomic profile for floral differentiation has been adopted in different species such as *Ranunculus glacialis* [60], *Chrysanthemum morifolium* [61], *Chrysanthemum lavandulifolium* [62], *Staphisagria Ranunculaceae* [63], rice [64], and Delphinieae [63]. The obtained results in this study suggested significant variation in the expression profiles at the different stages.

We identified putative genes responsible for floral differentiation in *E. japonicum* and observed upregulated expression of *ELF3*, *PHD*, *cullin1*, *SE14*, *ZSWIM3*, *GIDNATEA*, and *SERPIN B* from the initial primordium differentiation stage to the perianth differentiation stage. *ELF3* plays a crucial role in regulating the circadian clock and is responsible for many downstream regulatory pathways [65]. Furthermore, *ELF3* interacts with *ELF4*, *LUX*, and other proteins to regulate hypocotyl extension, thermo-morphogenesis, and flowering time [66, 67]. In addition, *ELF3* gene has been reported to suppress cell elongation under increasing temperature [65]. Thus, further functional analysis of *ELF3* can provide valuable insights into its role in regulating floral differentiation in *E. japonicum*. Similarly, other DEGs identified with differential expression at early stages of floral differentiation, including *PHD* [68–70], *cullin 1* [71], *SE14* [72], *ZSWIM3* [73], *GIGNATEA* [74], and *SERPIN B* [75], have been characterized for their positive role in the regulation of flowering in different plant species with their potential involvement in circadian pathways.

Comparative transcriptomic profile suggested that expression of *ELF3* and *FT*, *cullin 1*, *GLP1*, and *CONSTANS* significantly increased at later stages, suggesting an enhanced role in later flower differentiation stages, viz., stamen differentiation, and pistil differentiation. Similarly, stage-specific genes were identified for each transition stage during floral organ development. Similar results have been reported previously, suggesting stage-specific regulatory genes [76, 77]. *MADS* domain protein APETALA1 (*AP1*) and LEAFY (*LFY*) are generally considered as master regulators of flowering in plants [78]; however, activation of these genes is dependent on MADS domain proteins, including SUPPRESSOR OF OVEREXPRESSION OF CONSTANS 1 (*SOC1*), FRUITFULL (*FUL*), and AGAMOUS-LIKE 24 (*AGL24*) [79, 80].

TFs and their roles in the developmental process have been extensively studied over the past decades [81–83]. Several TFs such as *AP2/ERF*, *MYB*, *bHLH*, *MADS-box*, and *NAC* have been previously characterized for their active role in development and flower initiation in plants. Our study identified TFs such as *AP2/ERF* [84], *bHLH* [85], *FAR1* [86], *mTERF* [87], *MYB-related* [88], *NAC* [89], *Tify* [90], and *WRKY* [91] as major regulators involved in floral differentiation in *E. japonicum*. Ethylene-Responsive Factor (*ERF*) gene family is known for its diverse role in plant developmental process, including germination, flowering, maturation, and senescence [92, 93]. Ethylene has been reported with its regulatory role in the transition to flowering phase [94, 95], and ethylene regulation in flowering plants is controlled by ERF gene family [96]. The *bHLH* TF family regulates *CONSTANS* in Arabidopsis, which is crucial for photoperiodic flowering. Similarly, *FAR1*, an important regulator in the photo-sensitive circadian clock, regulates *ELF4* by directly binding to *FBS* cis-elements and promotes flowering [97]. However, the flowering phase in *E. japonicum* starts underground in the absence of light. Therefore, further characterization of *bHLH* and FAR1 and their relationship with *CONSTANS* in *E. japonicum* may yield a potential breakthrough in activating flower organs of underground bulbs in ephemeral plants. Myb-related protein positively regulates flowering by activating FLOWERING LOCUS T and FLOWERING LOCUS T INTERACTING PROTEIN 1 [88]. Furthermore, based on GO terms associations, we identified twelve upregulated TFs at the four stages of flower initiation. Therefore, we speculated that these TFs play a crucial role in underground floral differentiation in *E. japonicum.*

## 5. Conclusions

This study investigated the transcriptional profiles of underground floral differentiation in *E. japonicum* at four developmental stages. Through a comparative transcriptome analysis, we identified several putative candidate genes, including *ELF3*, *PHD*, *cullin 1*, *SE14*, *ZSWIM3*, *GIGNATEA*, *SERPIN B*, *bHLH*, *FAR1*, *mTERF*, *MYB-related*, *NAC*, *Tify*, and *WRKY*. Further functional characterization of these putative candidate genes can provide a better understanding of the process of underground floral organ differentiation in *E. japonicum.* Furthermore, the excavated information can be used as a base study for further characterization of floral differentiation in spring ephemeral plants.

## Data Availability

The raw RNA-seq data has been submitted to NCBI SRA under the project number: PRJNA730644.

## Abbreviations

DEG: Differentially expressed gene
FPKM: Fragments per kilobase of exon model per million reads mapped
FRD: False discovery rate
GO: Gene ontology
KEGG: Kyoto Encyclopedia of Genes and Genomes
PCA: Principal component analysis
qRT-PCR: Quantitative real-time PCR
TF: Transcription factor.

## Supplementary Materials

Supplementary Materials Figure S1: annotation of unigenes from different databases; Table S1: list of primers corresponding to selected DEGs used for qRT-PCR; Table S2: statistics of transcriptome sequencing; Table S3: list of differentially expressed genes corresponding to different floral developmental stages; Table S4: differentially expressed genes related to floral differentiation identified in comparison of Az (flower primordium differentiation) and Bz (perianth differentiation); Table S5: differentially expressed genes identified related to floral differentiation in comparison of Az (flower primordium differentiation) and Cz (stamen differentiation); Table S6: differentially expressed genes identified related to floral differentiation in comparison of Az (flower primordium differentiation) and Dz (pistil differentiation); Table S7: differentially expressed genes related to floral differentiation identified in comparison of Bz (perianth differentiation) and Cz (stamen differentiation); Table S8: differentially expressed genes related to floral differentiation identified in comparison of Bz (perianth differentiation) and Dz (pistil differentiation); Table S9: differentially expressed genes related to floral differentiation identified in comparison of Cz (stamen differentiation) and Dz (pistil differentiation); Table S10: differentially expressed transcription factors between different groups; Table S11: differentially expressed TFs associated with floral differentiation at different floral developmental stage.

## References

[1] Park J., Kim Y. T. (2020). Erythronium japonicum alleviates inflammatory pain by inhibiting MAPK activation and by suppressing NF-*κ*B activation via ERK/Nrf2/HO-1 signaling pathway. *Antioxidants*.

[2] Kondo T., Okubo N., Miura T., Honda K., Ishikawa Y. (2002). Ecophysiology of seed germination in Erythronium japonicum (Liliaceae) with underdeveloped embryos. *American Journal of Botany*.

[3] Ishii H., Sakai S. (2000). Optimal timing of corolla abscission: experimental study onErythronium japonicum(Liliaceae). *Functional Ecology*.

[4] Kim S. Y., Lee S. Y., Rhie Y. H., Kim K. S. (2014). Breaking bud dormancy in Erythronium japonicum Decne.(Liliaceae) by natural and artificial chilling. *Horticulture, Environment, and Biotechnology*.

[5] Baskin C. C., Baskin J. M. (2004). Germinating seeds of wildflowers, an ecological perspective. *HortTechnology*.

[6] Takada T., Nakayama S., Kawano S. (1998). A sensitivity analysis of the population dynamics of Erythronium japonicum, a liliaceous perennial. *Plant Species Biology*.

[7] Yoshie F., Fukuda T. (1994). Effects of growth temperature and winter duration on leaf phenology of Erythronium japonicum, a forest spring geophyte. *Oecologia*.

[8] Kawano S. (2005). Life-history monographs of Japanese plants. 1: Erythronium japonicum Decne. (Liliaceae). *Plant Species Biology*.

[9] Hui W., Yang Y., Wu G., Peng C., Chen X., Zayed M. Z. (2017). Transcriptome profile analysis reveals the regulation mechanism of floral sex differentiation in *Jatropha curcas* L. *Scientific Reports*.

[10] Luo T., Zhang J., Khan M. N., Liu J., Xu Z., Hu L. (2018). Temperature variation caused by sowing dates significantly affects floral initiation and floral bud differentiation processes in rapeseed (*Brassica napus* L). *Plant Science*.

[11] Liu Y., Hao X., Lu Q. (2020). Genome-wide identification and expression analysis of flowering-related genes reveal putative floral induction and differentiation mechanisms in tea plant (*Camellia sinensis*). *Genomics*.

[12] Lu H., Klocko A. L., Brunner A. M. (2019). RNA interference suppression of AGAMOUS and SEEDSTICK alters floral organ identity and impairs floral organ determinacy, ovule differentiation, and seed-hair development in Populus. *New Phytologist*.

[13] Higashiura M., Douzono M., Uno Y., Yamanaka M. (2021). Verification of the effects of end-of-day-cooling on floral differentiation and cut-flower characteristics in carnation (*Dianthus caryophyllus* L.). *The Horticulture Journal*.

[14] He W., Chen Y., Gao M. (2018). Transcriptome analysis of *Litsea cubeba* Floral buds reveals the role of hormones and transcription factors in the differentiation process. *G3: Genes, Genomes Genetics*.

[15] Han Y., Tang A., Yu J. (2019). RcAP1, a homolog of APETALA1, is associated with flower bud differentiation and floral organ morphogenesis in Rosa chinensis. *International Journal of Molecular Sciences*.

[16] Kurokawa K., Kobayashi J., Nemoto K. (2020). Expression of LhFT1, the flowering inducer of Asiatic hybrid lily, in the bulb scales. *Frontiers in Plant Science*.

[17] Quan S., Niu J., Zhou L., Xu H., Ma L., Qin Y. (2019). Stages identifying and transcriptome profiling of the floral transition in *Juglans regia*. *Scientific Reports*.

[18] Farré E. M., Harmer S. L., Harmon F. G., Yanovsky M. J., Kay S. A. (2005). Overlapping and Distinct Roles of *PRR7* and *PRR9* in the *Arabidopsis* Circadian Clock. *Current Biology*.

[19] Liu F., Wang Y., Ding Z. (2017). Transcriptomic analysis of flower development in tea (*Camellia sinensis* (L.)). *Gene*.

[20] Xia E.-H., Zhang H.-B., Sheng J. (2017). The tea tree genome provides insights into tea flavor and independent evolution of caffeine biosynthesis. *Molecular Plant*.

[21] Horvath D. P., Anderson J. V., Chao W. S., Foley M. E. (2003). Knowing when to grow: signals regulating bud dormancy. *Trends in Plant Science*.

[22] Horvath D. (2009). Common mechanisms regulate flowering and dormancy. *Plant Science*.

[23] Chen M., MacGregor D. R., Dave A. (2014). Maternal temperature history activates flowering locus T in fruits to control progeny dormancy according to time of year. *Proceedings of the National Academy of Sciences*.

[24] Nazir M. F., Jia Y., Ahmed H. (2020). Genomic insight into differentiation and selection sweeps in the improvement of upland cotton. *Plants*.

[25] Yu T., Zhang J., Cao J. (2021). Leaf transcriptomic response mediated by cold stress in two maize inbred lines with contrasting tolerance levels. *Genomics*.

[26] Hennig L. (2007). Patterns of beauty - omics meets plant development. *Trends in Plant Science*.

[27] Milyaev A., Kofler J., Klaiber I. (2021). Toward systematic understanding of flower bud induction in apple: a multi-omics approach. *Frontiers in Plant Science*.

[28] Lin K., Kools H., de Groot P. J. (2011). MADMAX–management and analysis database for multiple~ omics experiments. *Journal of Integrative Bioinformatics*.

[29] Agrawal P. K., Babu B. K., Saini N. (2015). Omics of model plants. *PlantOmics: The Omics of Plant Science*.

[30] Biswas P., Chakraborty S., Dutta S., Pal A., Das M. (2016). Bamboo flowering from the perspective of comparative genomics and transcriptomics. *Frontiers in Plant Science*.

[31] Samarth, Lee R., Song J., Macknight R. C., Jameson P. E. (2019). Identification of flowering-time genes in mast flowering plants using de novo transcriptomic analysis. *PLoS One*.

[32] Nejat N., Ramalingam A., Mantri N. (2018). Advances in transcriptomics of plants. *Plant genetics and molecular biology*.

[33] Hori K., Matsubara K., Yano M. (2016). Genetic control of flowering time in rice: integration of Mendelian genetics and genomics. *Theoretical and Applied Genetics*.

[34] Higgins J. A., Bailey P. C., Laurie D. A. (2010). Comparative genomics of flowering time pathways using Brachypodium distachyon as a model for the temperate grasses. *PLoS One*.

[35] Karsai I., Szűcs P., Kőszegi B. (2008). Effects of photo and thermo cycles on flowering time in barley: a genetical phenomics approach. *Journal of Experimental Botany*.

[36] Ubbens J. R., Stavness I. (2017). Deep plant phenomics: a deep learning platform for complex plant phenotyping tasks. *Frontiers in Plant Science*.

[37] Takáč T., Pechan T., Šamaj J. (2011). Differential proteomics of plant development. *Journal of Proteomics*.

[38] De-la-Peña C., Badri D. V., Lei Z. (2010). Root Secretion of Defense-related Proteins Is Development-dependent and Correlated with Flowering Time. *Journal of Biological Chemistry*.

[39] Yuan Z., Gao S., Xue D.-W. (2009). RETARDED PALEA1 controls PALEA development and floral zygomorphy in rice. *Plant Physiology*.

[40] Kater M. M., Dreni L., Colombo L. (2006). Functional conservation of MADS-box factors controlling floral organ identity in rice and Arabidopsis. *Journal of Experimental Botany*.

[41] Vining K. J., Romanel E., Jones R. C. (2015). The floral transcriptome ofEucalyptus grandis. *New Phytologist*.

[42] Fan Z., Li J., Li X. (2015). Genome-wide transcriptome profiling provides insights into floral bud development of summer-flowering *Camellia azalea*. *Scientific Reports*.

[43] Weigel D., Alvarez J., Smyth D. R., Yanofsky M. F., Meyerowitz E. M. (1992). *LEAFY* controls floral meristem identity in Arabidopsis. *Cell*.

[44] Irish V. F., Sussex I. M. (1990). Function of the apetala-1 gene during Arabidopsis floral development. *The Plant Cell*.

[45] Liu X., Yang Y., Zhu J., Qin J., Sun Z., Zhang L. (2018). Study on morphological differentiation and growth rhythm of underground buds of spring ephemeroid plant Erythronium japonicum during summer dormancy. *Journal of Nanjing Forestry University (Natural Sciences Edition)*.

[46] Grabherr M. G., Haas B. J., Yassour M. (2011). Full-length transcriptome assembly from RNA-Seq data without a reference genome. *Nature Biotechnology*.

[47] Noman A., Aqeel M., Deng J., Khalid N., Sanaullah T., Shuilin H. (2017). Biotechnological advancements for improving floral attributes in ornamental plants. *Frontiers in Plant Science*.

[48] Anders S., Huber W. (2012). *Differential Expression of RNA-Seq Data at the Gene Level–The DESeq Package*.

[49] Heberle H., Meirelles G. V., da Silva F. R., Telles G. P., Minghim R. (2015). InteractiVenn: a web-based tool for the analysis of sets through Venn diagrams. *BMC Bioinformatics*.

[50] Harris M. A., Clark J., Ireland A. (2004). The gene ontology (GO) database and informatics resource. *Nucleic Acids Research*.

[51] Kanehisa M., Goto S. (2000). KEGG: Kyoto encyclopedia of genes and genomes. *Nucleic Acids Research*.

[52] Zheng Y., Jiao C., Sun H. (2016). iTAK: a program for genome-wide prediction and classification of plant transcription factors, transcriptional regulators, and protein kinases. *Molecular Plant*.

[53] Livak K. J., Schmittgen T. D. (2001). Analysis of Relative Gene Expression Data Using Real-Time Quantitative PCR and the 2^−*ΔΔ* _C_^_T_ Method. *Methods*.

[54] Mortazavi A., Williams B. A., McCue K., Schaeffer L., Wold B. (2008). Mapping and quantifying mammalian transcriptomes by RNA-Seq. *Nature Methods*.

[55] Srikanth A., Schmid M. (2011). Regulation of flowering time: all roads lead to Rome. *Cellular and Molecular Life Sciences*.

[56] Kanno A. (2016). Molecular mechanism regulating floral architecture in monocotyledonous ornamental plants. *The Horticulture Journal*.

[57] Littlejohn G. The challenges of breeding wild flower cultivars for use in commercial floriculture: African Proteaceae.

[58] Kim H. J., Jung J. B., Jang Y. L., Sung J. H., Park P. S. (2015). Effects of experimental early canopy closure on the growth and reproduction of spring ephemeral Erythronium japonicum in a montane deciduous forest. *Journal of Plant Biology*.

[59] Álvarez-Buylla E. R., Chaos Á., Aldana M. (2008). Floral morphogenesis: stochastic explorations of a gene network epigenetic landscape. *PLoS One*.

[60] Mauracher S., Wagner J. (2021). Flower preformation in the nival plant Ranunculus glacialis L.: shoot architecture and impact of the growing season length on floral morphogenesis and developmental dynamics. *Alpine Botany*.

[61] Ding L., Zhao K., Zhang X. (2019). Comprehensive characterization of a floral mutant reveals the mechanism of hooked petal morphogenesis in Chrysanthemum morifolium. *Plant Biotechnology Journal*.

[62] Wen X., Qi S., Yang L., Hong Y., Dai S. (2019). Expression pattern of candidate genes in early capitulum morphogenesis of *Chrysanthemum lavandulifolium*. *Scientia Horticulturae*.

[63] Zalko J., Frachon S., Morel A. (2021). Floral organogenesis and morphogenesis of Staphisagria (Ranunculaceae): Implications for the evolution of synorganized floral structures in Delphinieae. *International Journal of Plant Sciences*.

[64] Duan Y., Chen Y., Li W. (2019). RETINOBLASTOMA-RELATEDGenes specifically control inner floral organ morphogenesis and pollen development in rice. *Plant Physiology*.

[65] Zhao H., Xu D., Tian T. (2021). Molecular and functional dissection of EARLY-FLOWERING 3 (ELF3) and ELF4 in _Arabidopsis_. *Plant Science*.

[66] Nusinow D. A., Helfer A., Hamilton E. E. (2011). The ELF4-ELF3-LUX complex links the circadian clock to diurnal control of hypocotyl growth. *Nature*.

[67] McWatters H. G., Bastow R. M., Hall A., Millar A. J. (2000). The *ELF3 zeitnehmer* regulates light signalling to the circadian clock. *Nature*.

[68] Qian F., Zhao Q. Y., Zhang T. N. (2021). A histone H3K27me3 reader cooperates with a family of PHD finger-containing proteins to regulate flowering time in Arabidopsis. *Journal of Integrative Plant Biology*.

[69] Greb T., Mylne J. S., Crevillen P. (2007). The PHD Finger Protein VRN5 Functions in the Epigenetic Silencing of *Arabidopsis FLC*. *Current Biology*.

[70] Matsubara K., Yamanouchi U., Nonoue Y. (2011). Ehd3, encoding a plant homeodomain finger-containing protein, is a critical promoter of rice flowering. *The Plant Journal*.

[71] Cheng W., Yin S., Tu Y., Mei H., Wang Y., Yang Y. (2020). SlCAND1, encoding cullin-associated Nedd8-dissociated protein 1, regulates plant height, flowering time, seed germination, and root architecture in tomato. *Plant Molecular Biology*.

[72] Yokoo T., Saito H., Yoshitake Y. (2014). Se14, encoding a JmjC domain-containing protein, plays key roles in long-day suppression of rice flowering through the demethylation of H3K4me3 of RFT1. *PLoS One*.

[73] Zhang Z., Zhang Z., Han X. (2020). Specific response mechanism to autotoxicity in melon (*Cucumis melo* L.) root revealed by physiological analyses combined with transcriptome profiling. *Ecotoxicology and Environmental Safety*.

[74] Park D. H., Somers D. E., Kim Y. S. (1999). Control of circadian rhythms and photoperiodic flowering by the Arabidopsis GIGANTEA gene. *Science*.

[75] Roberts T. H., Marttila S., Rasmussen S. K., Hejgaard J. (2003). Differential gene expression for suicide-substrate serine proteinase inhibitors (serpins) in vegetative and grain tissues of barley. *Journal of Experimental Botany*.

[76] Feng N., Song G., Guan J. (2017). Transcriptome profiling of wheat inflorescence development from spikelet initiation to floral patterning identified stage-specific regulatory genes. *Plant Physiology*.

[77] Yan W., Chen D., Schumacher J. (2019). Dynamic control of enhancer activity drives stage-specific gene expression during flower morphogenesis. *Nature Communications*.

[78] Wang J.-W., Czech B., Weigel D. (2009). miR156-Regulated SPL Transcription Factors Define an Endogenous Flowering Pathway in *Arabidopsis thaliana*. *Cell*.

[79] Lee J., Oh M., Park H., Lee I. (2008). SOC1 translocated to the nucleus by interaction with AGL24 directly regulates LEAFY. *The Plant Journal*.

[80] Lucero L. E., Manavella P. A., Gras D. E., Ariel F. D., Gonzalez D. H. (2017). Class I and class II TCP transcription factors modulate SOC1-dependent flowering at multiple levels. *Molecular Plant*.

[81] Dubos C., Stracke R., Grotewold E., Weisshaar B., Martin C., Lepiniec L. (2010). MYB transcription factors in *Arabidopsis*. *Trends in Plant Science*.

[82] Rushton P. J., Somssich I. E., Ringler P., Shen Q. J. (2010). WRKY transcription factors. *Trends in Plant Science*.

[83] Riechmann J. L., Ratcliffe O. J. (2000). A genomic perspective on plant transcription factors. *Current Opinion in Plant Biology*.

[84] Saidi A., Hajibarat Z. (2020). Computational study of environmental stress-related transcription factor binding sites in the promoter regions of maize auxin response factor (ARF) gene family. *Notulae Scientia Biologicae*.

[85] Liu Y., Li X., Li K., Liu H., Lin C. (2013). Multiple bHLH proteins form heterodimers to mediate CRY2-dependent regulation of flowering-time in Arabidopsis. *PLoS Genetics*.

[86] Xie Y., Zhou Q., Zhao Y. (2020). FHY3 and FAR1 Integrate Light Signals with the miR156-SPL Module-Mediated Aging Pathway to Regulate *Arabidopsis* Flowering. *Molecular Plant*.

[87] Wobbe L. (2021). The molecular function of plant mTERFs as key regulators of organellar gene expression. *Plant and Cell Physiology*.

[88] Abe M., Kaya H., Watanabe-Taneda A. (2015). FE, a phloem-specific Myb-related protein, promotes flowering through transcriptional activation of FLOWERING LOCUS T and FLOWERING LOCUS T INTERACTING PROTEIN 1. *The Plant Journal*.

[89] Yoo S. Y., Kim Y., Kim S. Y., Lee J. S., Ahn J. H. (2007). Control of flowering time and cold response by a NAC-domain protein in Arabidopsis. *PLoS One*.

[90] Guan Y., Ding L., Jiang J. (2021). Overexpression of the *CmJAZ1-like* gene delays flowering in Chrysanthemum *morifolium*. *Horticulture research*.

[91] Li W., Wang H., Yu D. (2016). *Arabidopsis* WRKY Transcription Factors WRKY12 and WRKY13 Oppositely Regulate Flowering under Short-Day Conditions. *Molecular Plant*.

[92] Liu J., Li J., Wang H., Fu Z., Liu J., Yu Y. (2011). Identification and expression analysis of ERF transcription factor genes in petunia during flower senescence and in response to hormone treatments. *Journal of Experimental Botany*.

[93] Liu M., Sun W., Ma Z. (2019). Genome-wide investigation of the AP2/ERF gene family in tartary buckwheat (*Fagopyum Tataricum*). *BMC Plant Biology*.

[94] Ogawara T., Higashi K., Kamada H., Ezura H. (2003). Ethylene advances the transition from vegetative growth to flowering in *Arabidopsis thaliana*. *Journal of Plant Physiology*.

[95] Reid M. S., Wu M.-J. (1992). Ethylene and flower senescence. *Plant Growth Regulation*.

[96] Li X., Zhu X., Mao J. (2013). Isolation and characterization of ethylene response factor family genes during development, ethylene regulation and stress treatments in papaya fruit. *Plant Physiology and Biochemistry*.

[97] Ma L., Li G. (2018). FAR1-related sequence (FRS) and FRS-related factor (FRF) family proteins in Arabidopsis growth and development. *Frontiers in Plant Science*.

