# Supplementary material for: Transcriptomic Insight into Underground Floral Differentiation in Erythronium japonicum
Source: Biomed Res Int. 2022 Jan 18;2022:4447472. doi: 10.1155/2022/4447472 (PMC8789427; doi:10.1155/2022/4447472)
Supplement: Supplementary Materials — Figure S1: annotation of unigenes from different databases; Table S1: list of primers corresponding to selected DEGs used for qRT-PCR; Table S2: statistics of transcriptome sequencing; Table S3: list of differentially expressed genes corresponding to different floral developmental stages; Table S4: differentially expressed genes related to floral differentiation identified in comparison of Az (flower primordium differentiation) and Bz (perianth differentiation); Table S5: differentially expressed genes identified related to floral differentiation in comparison of Az (flower primordium differentiation) and Cz (stamen differentiation); Table S6: differentially expressed genes identified related to floral differentiation in comparison of Az (flower primordium differentiation) and Dz (pistil differentiation); Table S7: differentially expressed genes related to floral differentiation identified in comparison of Bz (perianth differentiation) and Cz (stamen differentiation); Table S8: differentially expressed genes related to floral differentiation identified in comparison of Bz (perianth differentiation) and Dz (pistil differentiation); Table S9: differentially expressed genes related to floral differentiation identified in comparison of Cz (stamen differentiation) and Dz (pistil differentiation); Table S10: differentially expressed transcription factors between different groups; Table S11: differentially expressed TFs associated with floral differentiation at different floral developmental stage. [file 4447472.f1.zip › Table S1 (1).pdf]

Table S1. List of primers corresponding to selected DEGDs u

Gene ID	Name
Cluster-35905.71088	ELF3
Cluster-35905.51067	PHD
Cluster-35905.55785	cullin 1
Cluster-35905.53224	SE14
Cluster-35905.46244	ZSWIM3
Cluster-35905.88176	GIGNATEA
Cluster-35905.48750	SERPIN B
Cluster-35905.68695	bHLH
Cluster-35905.102970	FAR1
Cluster-35905.104427	mTERF
Cluster-31173.0	MYB-related
Cluster-35905.40908	NAC
Cluster-35905.86079	Tify
Cluster-35905.90345	WRKY
Cluster-38972.0	CONSTANS
Cluster-35905.60030	*AtMBD9*
Cluster-47057.0	GATA-binding protein
Cluster-35905.48792	cinnamoyl-CoA reductase 1-like isofor
Actin7	

ısed for qRT-PCR

Forward Primer
CAAAGCCTTTACGCAAGGAG
TCGATGAATGGCAACGATAA
CATTTTGCCAGGTTCAAGGT
AGCCAGTGGATCCATTTCAC
CATGTCTGGCAACAAAATCG
TTGGCCATGTTGTCACTTGT
TAATTGGCCCCTTCAACAAG
TGGGTTCTATGGCAAAGGAT
AGTACGGCAAGGGACACATC
GCCCAAGCTTTAATGCCATA
TAGAGCCATGGACCGCTAAC
AACGCGAAACTTGAGATGCT
CACAAGGAATGGCTGACAGA
CGCAACAATTGACAAGGCTA
AACCACAATTTCCCCAACAA
TTTGGGATCCAAACTCTTCG
CCTGAGGAATTTGGACCTCA
TTGGTGAAGGTGAGCATGG
AGCAAGAAGCATGAAGTCCA

**Reverse Primer**
GTAAGCAAGTGGCGCATACA
TAACTCGTCCCGAAAACACC
TGGAACCTGCTACATTGCTG
GAAGCTCAGCAGCATCAGAA
GATAATGGCGAATGGCTGAT
ACGTGAGCAAACCCCTTTTT
ACAGCGTTCTGAAAGCAACC
GGATGGTCCTTGTCAAACTCA
CACTCACCCAGAATGGCTTT
TCATCCCTGGAATGTCGAGT
GATTACCACGTACAATCGGA
GACGTAAATTCACCGGCAGT
ACCACGACAATGTGATCGAA
GGAATATGCGATGGATCACC
TTGGACCACCTTCAACAAAG
CACCGCAATGTGAGTCATTC
ACCCATTCAAAGCATTTTGC
GTCCATCAGTCTCCCCTCAT
AAATTCTTCCTCAGCGCAAC
