# Supplementary material for: Transcriptomic Insight into Underground Floral Differentiation in Erythronium japonicum
Source: Biomed Res Int. 2022 Jan 18;2022:4447472. doi: 10.1155/2022/4447472 (PMC8789427; doi:10.1155/2022/4447472)
Supplement: Supplementary Materials — Figure S1: annotation of unigenes from different databases; Table S1: list of primers corresponding to selected DEGs used for qRT-PCR; Table S2: statistics of transcriptome sequencing; Table S3: list of differentially expressed genes corresponding to different floral developmental stages; Table S4: differentially expressed genes related to floral differentiation identified in comparison of Az (flower primordium differentiation) and Bz (perianth differentiation); Table S5: differentially expressed genes identified related to floral differentiation in comparison of Az (flower primordium differentiation) and Cz (stamen differentiation); Table S6: differentially expressed genes identified related to floral differentiation in comparison of Az (flower primordium differentiation) and Dz (pistil differentiation); Table S7: differentially expressed genes related to floral differentiation identified in comparison of Bz (perianth differentiation) and Cz (stamen differentiation); Table S8: differentially expressed genes related to floral differentiation identified in comparison of Bz (perianth differentiation) and Dz (pistil differentiation); Table S9: differentially expressed genes related to floral differentiation identified in comparison of Cz (stamen differentiation) and Dz (pistil differentiation); Table S10: differentially expressed transcription factors between different groups; Table S11: differentially expressed TFs associated with floral differentiation at different floral developmental stage. [file 4447472.f1.zip › Table S2 (1).pdf]

Table S2. Statistics of the transcriptome assembly

Sample	Raw Reads	Clean Reads	Clean Base(G)	Error Rate(%)	Q20(%)	Q30(%)	GC Content(%)
A1	48386362	45469068	6.82	0.02	98.27	94.98	49.29
A2	43573798	41483374	6.22	0.02	98.12	94.56	49.34
A3	51209956	48651228	7.3	0.03	97.6	93.12	48.35
B1	47237892	44742926	6.71	0.03	97.73	93.49	49.71
B2	43435214	40950476	6.14	0.03	97.4	92.81	49.94
B3	41962222	39834968	5.98	0.03	97.7	93.39	49.65
C1	47840048	45749066	6.86	0.03	97.73	93.48	49.63
C2	41551288	39759290	5.96	0.03	97.64	93.3	49.45
C3	43934096	42210420	6.33	0.03	97.78	93.56	49.56
D1	45295658	42992428	6.45	0.03	97.61	93.25	49.9
D2	48203716	45267396	6.79	0.03	97.59	93.25	50.11
D3	46241392	43180546	6.48	0.03	97.38	92.72	49.98

Type	Number	Mean length	N50
Transcript	263291	561	727
Unigene	178951	706	880
