# Supplementary figures and images for: Transcriptomic Insight into Underground Floral Differentiation in Erythronium japonicum
Source: Biomed Res Int. 2022 Jan 18;2022:4447472. doi: 10.1155/2022/4447472 (PMC8789427; doi:10.1155/2022/4447472)

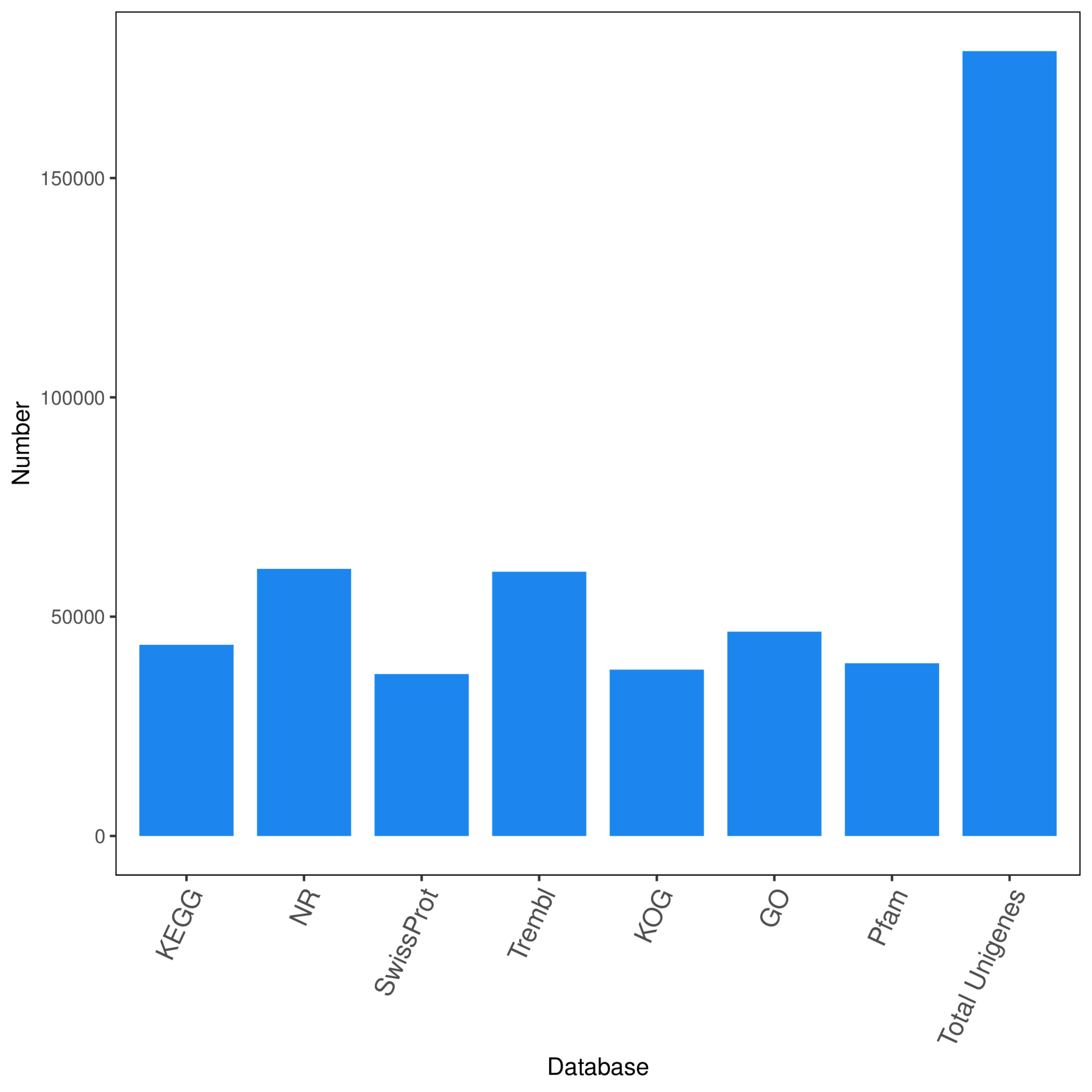

Supplement: Supplementary Materials — Figure S1: annotation of unigenes from different databases; Table S1: list of primers corresponding to selected DEGs used for qRT-PCR; Table S2: statistics of transcriptome sequencing; Table S3: list of differentially expressed genes corresponding to different floral developmental stages; Table S4: differentially expressed genes related to floral differentiation identified in comparison of Az (flower primordium differentiation) and Bz (perianth differentiation); Table S5: differentially expressed genes identified related to floral differentiation in comparison of Az (flower primordium differentiation) and Cz (stamen differentiation); Table S6: differentially expressed genes identified related to floral differentiation in comparison of Az (flower primordium differentiation) and Dz (pistil differentiation); Table S7: differentially expressed genes related to floral differentiation identified in comparison of Bz (perianth differentiation) and Cz (stamen differentiation); Table S8: differentially expressed genes related to floral differentiation identified in comparison of Bz (perianth differentiation) and Dz (pistil differentiation); Table S9: differentially expressed genes related to floral differentiation identified in comparison of Cz (stamen differentiation) and Dz (pistil differentiation); Table S10: differentially expressed transcription factors between different groups; Table S11: differentially expressed TFs associated with floral differentiation at different floral developmental stage. [file 4447472.f1.zip › Figure S1.pdf]
